# Dynamic performance of rotor-side nonlinear control technique for doubly-fed multi-rotor wind energy based on improved super-twisting algorithms under variable wind speed

**DOI:** 10.1038/s41598-024-55271-7

**Published:** 2024-03-07

**Authors:** Habib Benbouhenni, Mourad Yessef, Ilhami Colak, Nicu Bizon, Hossam Kotb, Kareem M. AboRas, Ali ELrashidi

**Affiliations:** 1 Department of Electrical & Electronics Engineering, Faculty of Engineering and Architecture, Nisantasi University, Istanbul, 34481742 Turkey; 2 LIMAS Laboratory, Faculty of Sciences Dhar El Mahraz, SMBA University, 30002 Fez, Morocco; 3 Pitești University Center, The National University of Science and Technology POLITEHNICA Bucharest, 110040 Pitesti, Romania; 4 ICSI Energy, National Research and Development Institute for Cryogenic and Isotopic Technologies, 240050 RamnicuValcea, Romania; 5 Department of Electrical Power and Machines, Alexandria University, Alexandria, 21544 Egypt; 6 Electrical Engineering Department, University of Business and Technology, 23435 Jeddah, Saudi Arabia; 7 Mathematical Engineering Department, Alexandria University, Alexandria, 21544 Egypt

**Keywords:** Multi-rotor wind energy, Rotor side converter, Dual super-twisting sliding mode command, Doubly-fed induction generator, Direct power control, Energy science and technology, Engineering

## Abstract

The paper proposes a nonlinear controller called dual super-twisting sliding mode command (DSTSMC) for controlling and regulating the rotor side converter (RSC) of multi-rotor wind power systems that use doubly-fed induction generators. It was proposed that this controller be developed as an alternative to the direct power control (DPC), which makes use of a pulse width modulation (PWM) strategy to regulate the RSC's functioning. Overcoming the power/current quality issue with the proposed technique (DPC-DSTSMC-PWM) is characterized by great robustness and excellent performance. The designed strategy was contrasted with the standard method of control and other methods already in use. So, the unique proposed control strategy’s robustness, performance, efficiency, and efficacy in enhancing system characteristics were tested and validated in Matlab/Simulink. In both tests, the proposed method resulted in significant improvements, reducing active power ripples by 83.33%, 57.14%, and 48.57% in the proposed tests. When compared with the traditional regulation method, the reduction rates of reactive power ripples are 64.06%, 52.47%, and 68.7% in the tests. However, in contrast to the conventional method, the proposed tests showed a decrease of between 72.46%, 50%, and 76.22% in the value of total harmonic distortion (THD) of the provided currents. These ratios show how effective the proposed plan is in ameliorating and enhancing aspects of the energy system.

## Introduction

Nowadays, in almost every field, electrical energy (EE) is widely used to operate devices and for industrial uses, which creates an increasing demand for it, as traditional sources have become insufficient and do not meet the increasing energy demand. All traditional sources adopted for generating EE cause several unwanted problems, such as the spread of toxic gases and environmental pollution^1^. Also, the use of conventional sources makes the bill for the production and consumption of EE high. Because of these problems and defects, other energy systems must be considered that do not depend on traditional sources and can provide a very large amount of EE to meet the increasing demand^2^. Because the EE generated from the sources is not of high quality at all, and the resulting current has ripples, these ripples affect the network and the energy system itself. The energy systems are numerous, and the difference between these systems lies in the sources used, as the sources used can be classified into renewable sources and non-renewable sources^3^. The latter is undesirable because it causes several defects and is costly. Therefore, renewable sources are considered one of the most prominent sources at present for producing EE, as a major shift to these sources is observed throughout the world as they have become a solution to several problems. The use of these sources contributes significantly to protecting the environment and minimizing the emission of toxic gases. Also, its use significantly reduces the cost of producing and consuming EE. Solar power is considered one of the most prominent renewable powers in the world, especially in desert areas where the temperature is very high, where this free energy available throughout the year can be exploited to generate electrical energy^4^. To exploit solar energy to generate EE, photovoltaic cells are used for this purpose, as these cells are used in the form of sheets. The issue of generating EE from solar energy has been addressed in several different scientific works^5^, where all of these works have proven the effectiveness of this energy and its importance in the energy and industrial fields. To exploit solar energy, farms are used for this purpose, as these farms contain thousands of photovoltaic cell panels, which require a vast area to generate the EE necessary to meet the city’s demand. The energy produced by the photovoltaic system is considered less efficient and with low quality of EE (there are ripples), so to exploit this energy well, filters must be used with the photovoltaic system. These filters have been discussed in several scientific works regarding the photovoltaic system and their importance in increasing EE quality^6^. In^7^, the author used a novel self-adaptive fuzzy-PID controller (SAFPIDC) to control the active filter. This strategy was relied upon to improve the quality of the current and minimize the value of total harmonic distortion (THD) of current compared to traditional control. The proposed control depends on experience as a result of the use of fuzzy logic (FL), as experimental and simulation results showed the effectiveness of the designed system in improving the quality of current and overcoming defects in traditional energy systems. Another work dealt with filters using hybrid particle swarm optimization-grey wolf optimization (PSO-GWO) and fractional-order proportional-integral-derivative controller (FOPIDC)^8^. In this work, the importance of filters and the proposed control in improving EE quality and reducing the value of THD of current was discussed. Experimental work was used to verify the validity of the proposed energy system. Experimental results show that the designed power system improves the quality of current and EE significantly, which is a good thing. In^9^, the author used bothdual-tree complex wavelet transform (DT-CWT), conventional proportional–integral (CPI), and type-1 and type-2 fuzzy logic controllers (T1FLC and T2FLC) in order to control an active filter to overcome the problem of low EE quality and current. The use of these strategies contributes significantly to increasing the EE quality and reducing the value of THD of current, and this is proven by the obtained experimental and simulation results. However, despite these solutions, the problem of EE quality remains, which is undesirable.

In addition to solar energy, wind energy (WE) is a renewable source that has proven its efficiency and great ability to meet the large demand for EE, where turbines called wind turbines are used^10^. These turbines convert WE into mechanical energy, and this energy gained from the wind is used to generate electrical current using generators^11^. In WE, a single turbine of large dimensions can be used to finance an entire city with EE, which is not possible in the case of solar energy^12^. The EE resulting from WE depends on both the wind speed and the size of the turbine, which contributes significantly to reducing costs and increasing the EE produced^13^. The use of wind turbines has both positives and negatives, the most prominent of which is that it reduces the costs of producing and consuming EE compared to non-traditional sources. Also, it contributes significantly to reducing the emission of toxic gases and protecting the environment from the risks of pollution, as the use of wind turbines does not contribute to the presence or production of dangerous materials^14^. One of its disadvantages is that it should not be placed in the path of bird migration, as it hinders the movement of birds and contributes to their elimination, which is undesirable. Also, wind turbines are very vulnerable to hurricanes and bad weather, causing damage and thus increasing costs^15^. In addition, when a fire occurs in the turbine, it cannot be extinguished due to the seriousness of the matter, and this contributes to increasing costs and reduces the lifespan of the turbine^16^. On the other hand, the use of traditional three-blade turbines or other types does not contribute significantly to increasing the energy gained from the wind. In wind farms, these turbines are affected by the wind generated between the turbines, as the farm’s yield as a whole decreases as a result of these winds, and therefore the amount of EE generated is affected, which is undesirable, especially during peak demand for it. Therefore, researchers developed these turbines and tried to overcome these defects and problems. A multi-rotor wind turbine (MRWT) system was proposed for this purpose. This new technology has been discussed in several scientific works^17–20^, where the mathematical model of this turbine was discussed, mentioning the negatives and positives.

Traditionally, MRWT is considered one of the most prominent technologies that have recently emerged for generating EE from WE, as it is not widespread because it is a very advanced technology and has a high cost^18^. The use of this type of turbine leads to overcoming the problems and defects found in traditional turbines, as the use of this technology leads to significantly overcoming the wind generated between the turbines in wind farms^19^. Also, its use leads to a significant increase in the energy gained from the wind compared to traditional turbines, as the energy is increased by an estimated 25% to 35%, which is a significant percentage of great importance in the field of renewable energies. The use of MRWT greatly increases the durability and stability of the energy system, as this technology is highly resistant to strong winds and hurricanes^21^. This technology relies on the use of several turbines of different dimensions in order to form the mother turbine, where the resulting energy is the sum of the energies of each turbine^22^. Using MRWT technologies requires more mechanical components and therefore regular maintenance, which increases costs, as this technology is considered expensive and expensive compared to traditional turbines, which is a negative^23^. Due to the use of several turbines, this technology is difficult to control, so the maximum power point tracking (MPPT) strategy is used for this purpose. So the MPPT strategy for MRWT is complex and difficult to achieve compared to MPPT for conventional turbines, as many equations are used to achieve the MPPT strategy^24^. In wind turbines, the energy gained from the wind is converted into electrical energy using generators. The latter are of several types, as direct current generators, synchronous machines, and asynchronous machines can be used for this purpose. The use of the generator must be compatible with the power generated by the turbine. For better operation, the power of the generator must be approximately the same as the power of the turbine. The doubly-fed induction generator (DFIG) is considered one of the most prominent and widely used types of generators in wind turbines on land and at sea due to several characteristics that distinguish this generator from other types^25^. Durability, low cost, ease of control, low maintenance, and greater efficiency and performance in variable wind speeds are the most prominent features of DFIG^26^. Also, the resulting energy can be controlled by feeding the rotating part of the generator, which allows for greater energy of high quality, as the stator of the generator is connected directly to the network without an intermediary, which is a feature not found in other generators. To feed the DFIG, two different inverters are used^27^. The inverter on the grid side is called the grid side converter (GSC), and its role is to convert alternating voltage to direct voltage, and the second inverter is called the rotor side converter (RSC), and its role is to convert constant voltage to alternating voltage. To control DFIG power, you must control both GSC and RSC, as two similar or different controls can be used for them. In most scientific works, only the RSC is controlled, and an uncontrolled inverter is used in the GSC in order to simplify the system and reduce the overall cost. In the field of control, it has been proposed to use several control strategies to control DFIG power, the most prominent of which are linear controls (direct power control (DPC)^28^ and direct torque control (DTC)^29^), smart controls (FL technique^30^, neural networks (NNs)^31^, genetic algorithm (GA)^32^), non-linear controls (Sliding mode control (SMC)^33^, synergetic control (SC)^34^, passivity control^25^, predictive control^35^, backstepping control (BC)^36^, super-twisting control (STC)^37^), and hybrid controls (FL-GA technique^38^, NN-FL-SMC technique^39^, DTC-STC-NN technique^40^, SMC-FL technique^41^, STC-SC technique^42^). These controls were mostly used to control the RSC, as they provided satisfactory results despite the presence of problems and defects. This is normal due to the nature of the turbine-based energy system. It is a nonlinear system, and with continuous and permanent operation, a change occurs in the parameter values, which affects the power quality. The influence of power quality on internal and external factors of the studied energy system is an undesirable matter that affects the network, causing disturbances in the operation of other devices and the energy system itself.

One of the linear strategies is the DPC technique, that has appeared in recent years as a genuine solution among the suggested solutions for controlling and regulating electrical machines. The DTC technique and this command scheme are extremely similar, as it has the same principle and idea, and the difference between them consists in the references used or the controlled quantities^43^. In the DPC technique, both the active (*Ps*) and reactive power (*Qs*) are controlled, while in the DTC technique, the torque and flux are controlled directly without the utilization of internal loops^44^. Simplicity, durability, low cost, and few gains are among the characteristics that the DPC technique is famous for compared to both vector control and field-oriented control (FOC)^45^. In the DPC technique, hysteresis comparator (HC) type controllers are used to control the power of the DFIG, where a three-level HC controller (1, 0, and − 1) is used to control the *Ps*. On the other hand, a two-level HC controller (1 and 0) is used to regulate the *Qs* of a DFIG-based wind turbine system. Their inputs are the resulting errors in both *Qs* and *Ps* of the machine.

In the DPC strategy of the DFIG, the estimation of both *Ps* and *Qs* is used to calculate the power error, where both voltage and current are measured. Estimating capabilities is linked first to estimating rotor flux, which makes the DPC strategy linked to the machine parameters, which is a negative thing that makes the DPC strategy affected in the event of a malfunction in the machine. However, some drawbacks limit the use of this technique, as the quality of the current is low and there are high ripples at the level of both the *Qs* and *Ps* as a result of using the hysteresis comparator ^46^. Also, a switching table (ST) is used to generate command pulses for the inverter IGBTs, which results in an electric current of non-constant frequency, which is undesirable^47^. In addition, the decrease in strategic durability is one of the most prominent of these undesirable defects resulting from the use of power estimation, as it causes an increase in the value of the power ripples and an increase in the THD value of the current. Several genuine solutions have been proposed and suggested to ameliorate the advantages and overcome the problems and defects of the DPC technique, such as the use of NN technique^48^, SC technique^49^, FL technique^50^, GA technique^51^, SMC strategy^52^, BC technique^53^, STC technique^54^, and fractional-order control^55^. Using these techniques leads to ameliorating the advantages and significantly reducing the *Qs* and *Ps* fluctuations. In^56^, the author combined fractional-order control and NN technique in order to overcome the disadvantages of the DPC strategy of DFIG-based MRWT, where the PWM strategy was used to control the operation of the RSC of DFIG-MRWT. A fractional-order NN controller was used to control the power, and the outputs of these controllers are the reference values of the voltage. The latter is used to generate the pulses necessary to operate the inverter. The advantage of this proposed strategy is that it is simple, more durable, has excellent performance, and is easy to implement. Also, it has few gains which makes it easy to adjust and change. This proposed strategy was implemented in a Matlab environment using several different tests running a 1.5 MW DFIG. The validity and safety of the proposed control and its efficiency in improving the characteristics of the energy system compared to the DPC strategy were verified in terms of the percentages of reduction in response time, ripples, THD value of the current, overshoot, and steady-state error (SSE) of DFIG power. In addition, the author made a comparison between the proposed strategy and some existing works, where the graphical and numerical results showed the superiority of this strategy over DPC and a group of works in terms of power quality and current. However, this strategy has a negative effect, which is the increase in the values of ripples and THD in the durability test as a result of using power estimation, which is an undesirable negative. In order to overcome the ill effects of low stream quality in the strategic DPC of DFIG-based wind turbine systems, different solutions exist; Including the use of fractional-order FL technique as a suitable and effective solution that has been relied upon in the work^57^. In the latter, the PWM strategy was used to generate the pulses necessary to operate the inverter. This strategy was relied upon to simplify control and reduce its total cost. Despite its many advantages, the use of the FL technique is not very effective in some circumstances and the quality of the stream is not significantly improved since there is no mathematical rule that shows how to use the FL strategy to obtain better results.

Researchers have developed new controls to overcome the drawbacks of the DPC strategy of DFIG, reduce power and current ripples, and overcome the drawbacks of traditional strategies. In^58^, the author proposed a controller of the form PI (1 + PI) in order to overcome the disadvantages of the DPC strategy of 1.5 MW DFIG-MRWT, where the PWM strategy was used to control the RSC. In this work, the MPPT strategy was used in order to obtain the reference value for the active power, and thus the value of the current and torque become related to the wind speed. The proposed strategy is characterized by the presence of a significant number of gains, which makes it difficult to adjust the dynamic response. Also, using capacity estimation, where the same rates are used in the traditional strategy, which contributes to raising the ripples and the THD of the current value in testing the durability or the occurrence of a malfunction in the system. Despite these shortcomings, the use of the PI (1 + PI) controller significantly improved the characteristics of the DPC strategy. Another solution, which is to use the integral SC technique in order to overcome the problems of the DPC of the DFIG strategy, was proposed in^59^. In this work, an integral SC technique control was used to control DFIG power. The proposed strategy is a change to the traditional strategy, as the author used the integral SC technique to compensate for the use of HC controllers and used the PWM strategy to replace the use of ST to generate operating pulses. The proposed control is simple, uncomplicated, easy to implement, has high durability, and has outstanding performance in reducing power ripples and increasing the quality of the current compared to the traditional strategy, and this is proven by the completed simulation results. The negative of this strategy lies in the use of power estimation, as a decrease in the quality of the current and an increase in the value of the energy ripples is observed, which is an undesirable negative. The SC and fractional-order control strategy were combined to overcome the disadvantages of the DPC strategy of a 1.5 MW DFIG-MRWT system^60^. The proposed fractional-order SC (FOSC) controller is characterized by simplicity, few gains, robustness, and ease of implementation. This proposed strategy plays the role of two different controllers, as it can be the DPC-SC technique or the DPC-FOSC technique. This proposed strategy was used to control the RSC only, with the MPPT strategy used to generate the reference value for the active power. The results obtained using the Matlab environment show the high performance of the proposed strategy compared to traditional control in terms of current/power quality, noting the presence of an increase in the ripple values for both current and torque in the event of changing the DFIG parameters, which is undesirable. A new strategy for DPC of DFIG is proposed as a suitable and effective solution to overcome the problem of ripples and low current quality^61^. In the latter, the modified SC technique was used in order to control the power with the use of the PWM strategy to generate control pulses in the RSC of a 1.5 MW DFIG-MRWT system. This proposed strategy is characterized by simplicity, low cost, fast dynamic response, small number of gains, outstanding performance, high durability, and ease of implementation. This proposed strategy was compared with the traditional strategy and some scientific works, where the necessary numerical and graphical results were extracted to accomplish this comparison, and the Matlab environment was used to obtain these results. The proposed strategy has a very fast dynamic response and presents fewer ripples compared to the proposed strategy and some scientific works, which is a positive thing. This strategy is negative in its behavior against changing machine parameters, as it is noted to be slightly affected compared to the traditional strategy and this appears through an increase in the value of THD of current and power ripples. In^62^, the author used a neural PI controller to control the powers of the DFIG-MRWT system, where the DPC strategy is used to control the machine. In this strategy, the modified space vector modulation (MSVM) technique was used to generate the pulses needed to operate the RSC of 1.5 MW DFIG. The DPC strategy based on neural PI controllers is a different strategy from the above-mentioned works and the traditional strategy, as it depends on estimating capabilities, and the same estimation equations found in the traditional strategy are used. The Matlab environment was used to implement this strategy, using several different tests to prove its effectiveness and ability to improve the characteristics of the studied energy system. The obtained numerical results show that the proposed strategy has a response time to capabilities that is much lower than the traditional strategy and some actions. The ripples, THD of current, SSE, and overshoot were also reduced by high percentages, which indicates the distinctive and effective performance of this strategy. PI controller and proportional derivative (PD) controller were used in the form of PD(1 + PI) to overcome the disadvantages of the DPC of 1.5 MW DFIG-MRWT strategy^63^, where the PWM strategy was used to convert the voltage reference values resulting from the PD(1 + PI) controller into pulses to turn on the RSC. The advantage of this strategy is simplicity and ease of implementation, as it is a change to the traditional strategy. Therefore, the power estimation process is used in this proposed strategy to obtain the power error, which makes it affected in the event of a malfunction in the machine. Another negative of this strategy lies in the high number of gains compared to the DPC strategy based on PI controllers, which is a negative matter that makes it difficult to control the dynamic response and this appears through the simulated results obtained, where it is noted that the proposed strategy has a longer response time than the traditional strategy. But in terms of ripple values, SSE, and overshoot, the proposed strategy provided better results compared to the traditional strategy, and this is shown by the relatively high reduction rates. The modified SMC technique is a new strategy that was proposed to overcome the problems of the SMC strategy, and it was used as a suitable solution^64^. In this work, the modified SMC strategy was used to control the power and reduce the current and torque ripples of the 1.5 MW DFIG-MRWT system, where the PWM is used to control the RSC. This proposed strategy has many advantages, such as simplicity, robustness, lack of precise knowledge of the mathematical model of the machine, ease of implementation, and the presence of a small number of gains, which makes it the most suitable for controlling capabilities. This proposed strategy was implemented in a Matlab environment, where a variable wind speed was used for this purpose. The graphical and numerical results show the robustness and ability of the DPC strategy based on the modified SMC technique to overcome the problem of low current quality and power ripples compared to the proposed strategy and some scientific papers. In the robustness test, it is noted that the DPC strategy based on the modified SMC technique is less affected compared to the traditional strategy, as the rates of influence were very low, which is a positive thing. However, the DPC technique based on these techniques becomes difficult to achieve, expensive more complex. Super-twisting SMC (STSMC) technique is among the non-linear methods designed and suggested to surpass some real problems of the SMC method, which is distinguished by high robustness, easy to apply, and can be used in many fields such as controlling electric machines and renewable powers^65^. In^66^, the author used the STSMC strategy to overcome the problems of the FOC technique of 1.5 MW DFIG. In this proposed strategy (FOC-STSMC), the PWM strategy based on the NN-FL algorithm was used to generate operating pulses for the RSC. The FOC-STSMC strategy is characterized by complexity and a significant number of gains compared to the traditional strategy and DPC. Despite this complexity, it provided satisfactory results, and this is shown by the simulation results in all completed tests. However, in the durability test, a noticeable increase in the ripple values is observed, which is negative. In^67^, the author used the STSMC strategy with the four-level neural MSVM strategy to improve the performance of the DPC strategy of the DFIG. In this proposed strategy, an STSMC strategy control was used to control the power, and power estimation was used for this purpose. The strategy provided satisfactory results compared to traditional control, as torque and current ripples were reduced. However, this strategy was proposed in the robustness test. It was noted that it was affected and this appears through the increase in energy ripples, which is negative. In the work^68^, the author made a comparison between the STSMC strategy and the SC technique in order to find out which strategy has the best ability to improve the performance of the DPC strategy. These controls were implemented using the Matlab environment, where a variable wind speed was used to accomplish this comparison. Through the graphical and numerical results, it is noted that the STSMC strategy provided unsatisfactory results compared to the SC strategy, which is negative. To overcome the problems of the STSMC strategy, several solutions have been proposed to improve the performance and effectiveness of this strategy. The neural STSMC strategy was used as an effective solution to improve the performance of the DPC strategy of DFIG and reduce the value of energy ripples^54,69^. The use of neural networks increased the performance and robustness of the STSMC strategy, and thus the performance of the DPC strategy. The negative of using neural networks is the lack of a mathematical rule for choosing the number of internal layers or the number of cells in each layer, which makes it difficult to determine the appropriate solution. In^70^, the author used the NN-FL algorithm to overcome the problems and defects of the STSMC strategy of the DPC technique, where the sign(u) function was replaced by the NN-FL algorithm. This proposed strategy was compared with the neural STSMC strategy of the DPC technique. The obtained simulation results prove the high performance of the STSMC-NN-FL technique compared to the STSMC-NN strategy in improving the characteristics of the DPC strategy and reducing the value of energy ripples. The negative of this strategy lies in the lack of mathematical rules that determine the number of FL rules and the number of neurons for each layer. Also, its reliance on capacity estimation makes it affected in the event of a malfunction in the system, which is undesirable.

In^71^, the author used both the seven-level neural MSVM technique and the STSMC-NN-FL controller to improve the characteristics of the DPC strategy of a 1.5 MW DFIG-based wind turbine. This proposed strategy is somewhat complex and expensive compared to the traditional strategy, as it relies heavily on artificial intelligence, which makes it not use the mathematical model of the machine and is less effective in the event of a malfunction. The advantage of this strategy is that it is more durable and has high performance in reducing power ripples and reducing the THD of current. The Matlab environment was used to implement this proposed strategy, and the results showed high performance and the extent of reducing energy ripples compared to the traditional strategy. This strategy has a negative side, which is that it is used to estimate capabilities, which makes it linked to the machine’s parameters, which is a negative thing in the event of a defect that contributes to an increase in undulations and a decrease in durability and performance. SC technique and STSMC strategy are combined to overcome the drawbacks of the DPC technique of the DFIG^42^. The resulting strategy (SC-STSMC) is characterized by high performance and is effective in reducing power ripples and increasing the quality of the current compared to the traditional strategy. In this work, an SC-STSMC control was used for power control, with the MSVM strategy used to generate the pulses necessary to operate the RSC of 1.5 MW DFIG. The advantage of this strategy is high performance and high efficiency in overcoming the problem of power ripples and significantly improving the quality of the current. The negatives of this strategy can be limited to the use of estimating capabilities and the presence of a significant number of gains. The simulation results obtained show high performance, although there is a noticeable effect on changing the machine parameters, as the ripple values ​​increased significantly, and this increase was less than the traditional strategy. Another strategy for STSMC has been proposed in^72^ in order to improve the properties of DPC of DFIG, which is represented by the fuzzy STSMC (FSTSMC) technique. The latter depends on experience and does not require precise knowledge of the mathematical model of the machine, as a control was used to control the capabilities. In addition to using the FSTSMC technique, the MSVM strategy was used to control the RSC of DFIG. This strategy is characterized by high performance and great durability compared to the traditional strategy. This strategy was implemented using the Matlab environment in different working conditions for DFIG, where the results obtained in all tests prove the superiority of the proposed strategy over traditional control in terms of improving the quality of current and power. This proposed strategy has disadvantages, which are that it is used to estimate capabilities and the lack of mathematical rules. It facilitates the application of the FL strategy. Also, this proposed strategy provided a longer potential time than the traditional strategy, which is undesirable. In^73^, the author proposed the use of fractional order control as a suitable solution to overcome the drawbacks and problems of the STSMC strategy. The proposed strategy is characterized by high durability and distinguished performance in improving the characteristics of the studied energy system. This proposed strategy is not related to the mathematical model of the system under study, which makes it provide better results. The Matlab environment was used to implement and verify this strategy, using several tests for this purpose. The simulation results show the high performance and robustness of the proposed strategy compared to the traditional strategy. In^74^, the author used the GA strategy to calculate the parameters of the STSMC controller used to improve the performance of the DPC strategy used in an energy system that contains a filter and a photovoltaic system. The proposed strategy is simple, easy to implement, and has distinctive and effective performance. This strategy was implemented in the Matlab environment, comparing the performance with the traditional strategy. The obtained graphical and numerical results demonstrate the high and distinctive performance of this strategy in improving the characteristics of the energy system compared to the traditional strategy. Another smart strategy proposed in^75^ to overcome the problems of the SMC strategy is Particle Swarm Optimization (PSO). The proposed strategy, represented by STSMC-PSO, was used to improve the characteristics of the indirect FOC technique of DFIG. This proposed strategy is characterized by very high performance and great durability against changing machine parameters, as the graphical and numerical results obtained from the simulation prove this matter. This proposed strategy was compared with the SMC strategy, where several different tests were used to compare them. All the results obtained show that the proposed strategy outperforms the SMC strategy in terms of improving the characteristics of the energy system. However, this proposed strategy has a negative side, which is the complexity and difficulty of implementation. Also, using power estimation, which is undesirable, contributes to raising the ripples. A new scheme of the fractional-order STSMC technique is proposed in^76^ to control a DFIG-based wind turbine system. This strategy is different and relies on the use of a new formula for fractional-order control, where using a gain that represents differential arithmetic, two different controls can be obtained, an advantage not found in other controls. This strategy is not complicated and does not use a mathematical model of the studied system, as the simulation was used to implement it and compare it with the traditional strategy. A variable wind speed was used to study the behavior of the proposed control. The numerical and graphical results showed the superiority of the proposed control over the traditional strategy in terms of improving the performance of the studied energy system. Fractional-order proportional-integral STSMC technique was proposed to overcome DFIG problems^77^. In this work, the PWM strategy was used to generate the pulses necessary to operate both RSC and GSC. The proposed control is characterized by high performance and great durability as a result of the use of a combination of various strategies and the use of the PSO strategy to calculate the values ​​of the gains of the proposed control. This proposed strategy has disadvantages that lie in complexity, difficulty of implementation, and the presence of a significant number of gains. Also, it is used for the estimation process, which contributes to raising the ripples and the THD value of the current in the durability test. This proposed strategy was implemented in a Matlab environment on a 1.5 MW generator under different working conditions, where different wind speeds were used. Numerical and graphical results extracted from the simulation demonstrate the superiority of the proposed control, but the undulations are not significantly overcome. The current quality is excellent in the proposed control compared to the conventional strategy. In this work, attention is paid to the STSMC controller because of its great importance in the field of control and its great ability to improve systems performance. A new strategy or idea will be given based on the use of the STSMC controller in order to control the DFIG-MRWT system.

In this work, a dual STSMC (DSTSMC) controller is utilized to ameliorate and improve the advantages and efficiency of the traditional DPC strategy of DFIGs, where two STSMC controllers are used in parallel to regulate the *Ps* and the same applies to the *Qs*. The proposed strategy differs from the traditional strategy (DPC) and the above-mentioned works in terms of principle and idea, as the MSVM strategy was used to generate the pulses necessary to operate the RSC of 1.5 MW DFIG-MRWT system. So, the essential contribution of the paper lies in proposing the DSTSMC technique as a suitable solution to reduce energy ripples and minimize the THD value of the current compared to the traditional DPC technique. Moreover, the second contribution lies in the use of the DPC-DSTSMC technique based on thetwo-level MSVM technique to regulate the DFIG-MRWT power. To demonstrate the efficiency of the suggested new control strategy in improving the system features, GSC is used using a diode, as the system becomes simpler and less expensive. The obtained method features high efficiency and low *Qs* and *Ps* ripples compared to the classical technique. Matlab software is used to verify the efficiency and performances of the DPC method based on the DSTSMC technique compared to the DPC-STSMC technique in terms of ripples reduction, THD value, robustness, reference tracking, and the SSE of *Qs* and *Ps* of the DFIG-based MRWT system. The objectives achieved by this work are explained in the following points:

- Significantly improving energy quality;
- Overcoming the shortcomings and problems of the DPC strategy;
- Reduction of THD values for current;
- Improving the performance and robustness of the STSMC strategy;
- Verifying the performance and effectiveness of the DSTSMC strategy;
- Using the MRWT turbine to generate electrical energy from wind;
- Increasing the durability of the energy system.

The article has been divided into five different sections. In the second section, an overview of the proposed strategy represented by the DSTSMC technique is given. The DPC-DSTSMC strategy is detailed in section “Two-level MSVM strategy”. Numerical and graphical results are given in section “Proposed DPC technique”, with two tests used for this purpose. In Ssection “Conclusions”, a summary of the work completed is mentioned, along with a mention of future work.

## Proposed DSTSMC strategy

STSMC technique is among the methods that are featured by high efficiency and high performance in enhancing systems' dynamic response. The STSMC technique is considered one of the nonlinear strategies that has shown distinctive performance and high robustness compared to linear controls and some controls, as using this strategy does not require precise knowledge of the mathematical model of the studied system. This strategy has fewer gains, which enables the dynamic response to be easily adjusted. Moreover, this strategy is easy to implement and inexpensive, as it can be easily applied to complex systems. This technique is a development of the SMC technique, which is specified by robustness, simplicity, and ease of execution compared to the SMC technique^65^. Equation (1) represents the STSMC technique used in this work.

1 $$\left\{ {\begin{array}{*{20}c} {\frac{{d\mathop u\nolimits_{1} }}{dt} = \mathop \alpha \nolimits_{2} {\text{sgn}} (S)} \\ {u = \mathop \alpha \nolimits_{1} \mathop {\left| S \right|}\nolimits^{r} {\text{sgn}} (S) + \mathop u\nolimits_{1} } \\ \end{array} } \right.$$

where, *α*_*1*_ and *α*_*2*_ are the gains of the STSMC controller,* S* is the surface; *r* is the exponent defined for the STSMC technique. In most applications, the value of *r* is taken the rang 0.3–0.7.

The STSMC controller has drawbacks, as its use does not significantly improve the characteristics of the studied energy system, which leads to thinking about effective solutions to overcome this problem. Several solutions were proposed as mentioned in works^42,54,69–77^, where all of these solutions provided additions and significantly improved the STSMC controller. But these solutions have positives and negatives, and most of the solutions used smart strategies, as these smart strategies depend on experience and there are no mathematical rules that facilitate their application, which is a negative thing. So, in this section, a simple and effective proposal is made that does not require knowledge of the mathematical model of the studied energy system.

The idea of the proposed solution is very simple and depends on using two STSMC techniques in parallel in order to increase the durability of the energy system and improve the performance of the DPC strategy. In order to obtain the mathematical model of the proposed controller, Eq. (1) is used for this purpose. In this proposed solution, there are no complex calculations and it can be accomplished easily, as it can be expressed by Eq. (2).

2 $$y\left( t \right) = \mathop y\nolimits_{1} (t) + \mathop y\nolimits_{2} (t)$$

With:

3 $$\left\{ \begin{gathered} y_{1} \left( t \right) = \alpha_{1} \mathop {\left( {\left| {S\left( t \right)} \right|} \right)}\nolimits^{{\mathop r\nolimits_{1} }} \cdot sign\left( {S\left( t \right)} \right) + \alpha_{2} \int {sign\left( {S\left( t \right)} \right)dt} \hfill \\ \mathop y\nolimits_{2} (t) = \alpha_{3} \mathop {\left( {\left| {S\left( t \right)} \right|} \right)}\nolimits^{{\mathop r\nolimits_{2} }} \cdot sign\left( {S\left( t \right)} \right) + \alpha_{4} \int {sign\left( {S\left( t \right)} \right)dt} \hfill \\ \end{gathered} \right.$$

where, *r*_*1*_ and* r*_*2*_* are* the exponents defined for the proposed DSTSMC technique. Therefore, the values of the exponents are *r*_*1*_ = 1/2 for *y*_*1*_*(t)* and *r*_*2*_ = 1/3 for the *y*_*2*_*(t)*.

α_1_, α_2_, α_3_, and α_4_ are the gains of proposed DSTSMC technique. Using these gains, the response of the proposed controller can be changed and modified. So, in this proposed controller, there are four gains, where smart strategies can be used to calculate their values.

**Figure 1:**
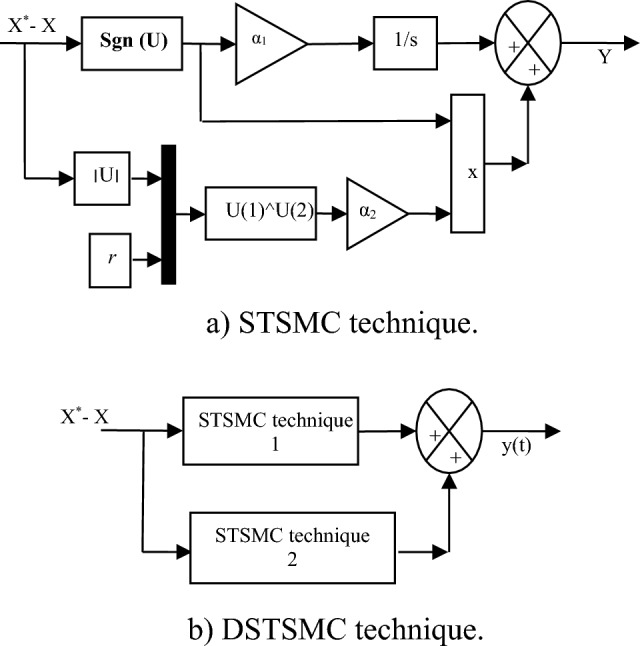
Proposed nonlinear controller.

Figure 1a represents the STSMC technique used to obtain the DSTSMC technique. Figure 1b represents a simple diagram of the proposed controller (DSTSMC) to improve and ameliorate the efficiency and capacity of the DPC-HC technique.

## Two-level MSVM strategy

Traditionally, the MSVM strategy is a new strategy that was proposed to overcome the shortcomings of the SVM strategy, which is the complexity and difficulty of implementation. It was proposed in the work^78^, mentioning its negatives and positives compared to both PWM and SVM techniques. This strategy was discussed in detail in the work^79^, where the necessary equations were given and applied to a two-level reflector. Moreover, it was performed experimentally using Dspace 1104, comparing the results obtained experimentally with the results of both PWM and SVM techniques. These experimental results show the high and effective performance of the MSVM strategy in terms of reducing the THD of current compared to both the PWM and SVM techniques.

This strategy depends on calculating both Maximum and Minimum for three-phase tensions, and then these values are combined to determine the corresponding times. In order to accomplish this strategy, the following main steps are followed^77,78^:

- Calculates the minimum voltages (min (*V*_*1*_, *V*_*2*_, and *V*_*3*_))
- Calculates the maximum voltages (max (*V*_*1*_, *V*_*2*_, and *V*_*3*_))
- Add the maximum and minimum voltages (max (*V*_*1*_, *V*_*2*_, and *V*_*3*_) + min (*V*_*1*_, *V*_*2*_, and *V*_*3*_)).
- The last step is to compare step-3 waveforms with *Vp*(V_Triangle_) and generate the pulses for that switch present in the 3-phase voltage source converter circuit.

**Figure 2:**
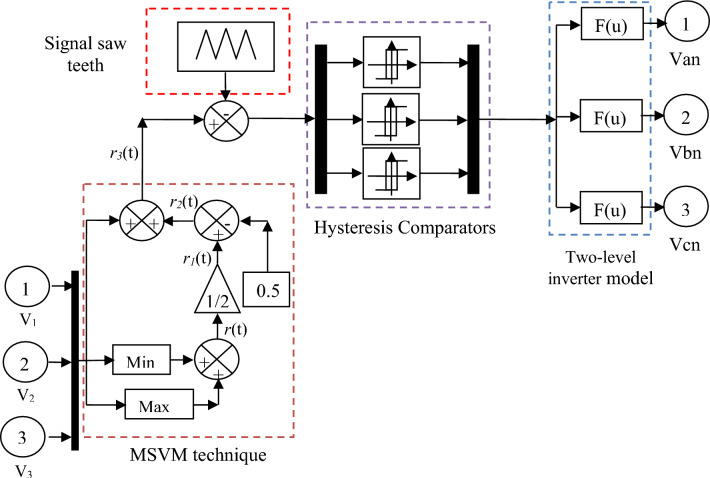
The two-level MSVM technique.

The two-level MSVM strategy used in this work is shown in Fig. 2.

Equation (4) represents the calculation of the Maximum and Minimum values of voltages.

4 $$r\left( t \right) = Max(\mathop V\nolimits_{1} ,\mathop V\nolimits_{2} ,and\mathop V\nolimits_{3} ) + Min(\mathop V\nolimits_{1} ,\mathop V\nolimits_{2} ,and\mathop V\nolimits_{3} )$$

From Eq. (4) the following equations can be extracted:

5 $$\mathop r\nolimits_{1} \left( t \right) = \frac{{Max(\mathop V\nolimits_{1} ,\mathop V\nolimits_{2} ,and\mathop V\nolimits_{3} ) + Min(\mathop V\nolimits_{1} ,\mathop V\nolimits_{2} ,and\mathop V\nolimits_{3} )}}{2}$$

6 $$\mathop r\nolimits_{2} \left( t \right) = \frac{{Max(\mathop V\nolimits_{1} ,\mathop V\nolimits_{2} ,and\mathop V\nolimits_{3} ) + Min(\mathop V\nolimits_{1} ,\mathop V\nolimits_{2} ,and\mathop V\nolimits_{3} )}}{2} - 0.5$$

The signal expressing this strategy is shown in the following equation:

7 $$\mathop r\nolimits_{3} \left( t \right) = \frac{{Max(\mathop V\nolimits_{1} ,\mathop V\nolimits_{2} ,and\mathop V\nolimits_{3} ) + Min(\mathop V\nolimits_{1} ,\mathop V\nolimits_{2} ,and\mathop V\nolimits_{3} )}}{2} - 0.5 + (\mathop V\nolimits_{1} ,\mathop V\nolimits_{2} ,and\mathop V\nolimits_{3} )$$

Based on Eq. (7), the pulses necessary to operate the RSC are generated, and this signal is compared with a signal from the Signal Saw Teeth in order to generate these pulses. So this strategy is simple and uncomplicated, and it was also accomplished experimentally in the work^80^ using the NNs technique, which provided very satisfactory results in terms of reducing the THD value of voltage and current. Due to its simplicity and ease of implementation, it was proposed to control the five-level inverter of the multi-phase permanent synchronous motor^81^. In this work, the MSVM strategy was used as a suitable solution to control a fifth-level inverter in order to reduce the complexity of the system and improve the value of THD of current, where the Matlab environment was used to implement the proposed system. The results obtained show that the value of THD of current has been significantly reduced while reducing the value of torque ripples, which is positive. In the works^82–84^, the MSVM strategy based on smart strategies was used, where all of the NNs, FL, and NN-FL techniques were used for this purpose in order to control the RSC of the DFIG-based wind turbine system. These smart strategies proposed for MSVM strategy have proven their high and effective performance compared to the traditional strategy. In these works, the pros and cons of the MSVM strategy were mentioned, and the Matlab environment was used to implement and verify these proposed strategies. Simulation results prove that applying these strategies leads to significantly improving the quality of the stream compared to other strategies. Therefore, this strategy can be relied upon in this work to obtain good results, as it is used in addition to the proposed controller to control capabilities and increase the quality of current/energy.

## Proposed DPC technique

**Figure 3:**
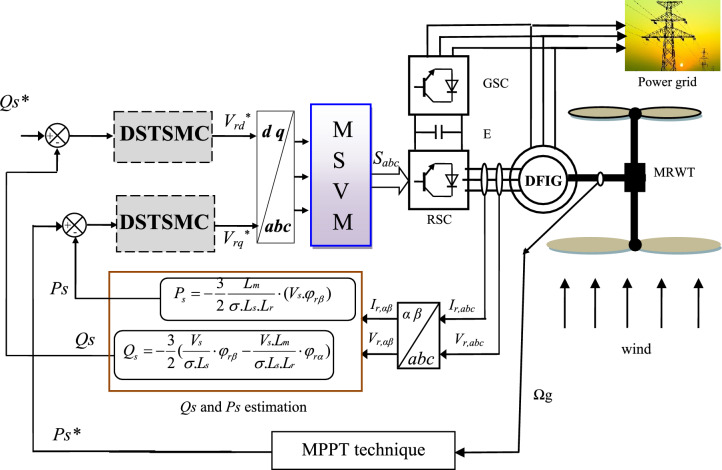
Proposed DPC technique of DFIG-MRWT.

In this section, an effective and simple solution is presented that relies on changing the traditional DPC strategy in order to overcome defects and problems, as this proposed solution is of great importance in the field of control. Figure 3 illustrates the new method suggested in this work for controlling the *Qs* and *Ps* of a 1.5 MWDFIG-based MRWT. The proposed nonlinear DP technique differs from the classical DPC strategy in principle and idea, where the HCs and ST were dispensed with and replaced with both the two-level MSVM technique and the suggested DSTSMC controller. In this strategy, two DSTSMC controllers are used to control the capabilities, as this controller has one input and one output. The inputs represent the power error and the voltage reference values represent the outputs. Therefore, the use of the DSTSMC controller aims to calculate the voltage reference values that are used to generate control pulses in the RSC. In this work, GSC is used with diodes to reduce the system complexity and overall cost. Also, to show the effectiveness and ability of the proposed strategy to improve the quality of current and energy. This proposed strategy has several advantages that distinguish it from the traditional strategy and control techniques mentioned above. One of the most important features of the proposed technique is its durability and high efficiency in reducing *Qs* and *Ps* ripples. Also, the quality of the supplied electric currents is improved compared to the traditional DPC method.

The suggested technique in this paper uses the same estimation equations utilized in the traditional DPC-HC technique to estimate both the *Qs* and *Ps*. In addition, the MPPT technique isutilizedand employed to calculate the reference value of the *Ps*. Also, the reference value of the *Qs* can be set to 0 VAR.

To estimate the DFIG energy, the Eqs. (8) and (9) are used. In addition, we need to know the rotor flux, where the voltage and current are measured to know and define the flux. Equation (10) is used to calculate and estimate the flux^51^.

8 $$P_{s} = - \frac{3}{2}\frac{Lm}{{\sigma \cdot Ls \cdot Lr}} \cdot (Vs \cdot \Psi_{r\beta } )$$

9 $$Q_{s} = - \frac{3}{2}\left( {\frac{Vs}{{\sigma \cdot Ls}} \cdot \Psi_{r\beta } - \frac{V \cdot Lm}{{\sigma \cdot Ls \cdot Lr}} \cdot \Psi_{r\alpha } } \right)$$

where, *Ψ*_*rβ*_ is the flux linkage of β-axis, *Lm* is the mutual inductance, *Ѱ*_*rα*_ is the rotor flux linkage of α-axis.

10 $$\left| {\overline{{\mathop \Psi \nolimits_{s} }} } \right| = \frac{{\left| {\overline{{\mathop V\nolimits_{s} }} } \right|}}{{\mathop w\nolimits_{s} }}$$

where, *V*_*s*_ is the voltage.

11 $$\sigma = 1 - \frac{{\mathop M\nolimits^{2} }}{{\mathop L\nolimits_{r} \mathop L\nolimits_{s} }}$$

Equation (12) can be used to calculate and estimate the quadrature and direct fluxes. The angle between *Ψ*_*sβ*_ and *Ψ*_*sα*_ is given by Eq. (14)^49,51^.

12 $$\left\{ {\begin{array}{*{20}c} {\mathop \Psi \nolimits_{s\alpha } = \int\limits_{0}^{t} {(\mathop V\nolimits_{s\alpha } } - \mathop R\nolimits_{s} \mathop I\nolimits_{s\alpha } )dt} \\ {\mathop \Psi \nolimits_{s\beta } = \int\limits_{0}^{t} {(\mathop V\nolimits_{s\beta } } - \mathop R\nolimits_{s} \mathop I\nolimits_{s\beta } )dt} \\ \end{array} } \right.$$

where, *V*_*sα*_ and *V*_*sβ*_ are the voltage linkage of α–β axis.

The flux is calculated and given by:

13 $$\mathop \Psi \nolimits_{s} = \sqrt {\mathop \Psi \nolimits_{s\alpha }^{2} + \mathop \Psi \nolimits_{s\beta }^{2} }$$

14 $$\mathop \theta \nolimits_{s} = arctg\left( {\frac{{\mathop \Psi \nolimits_{s\beta } }}{{\mathop \Psi \nolimits_{s\alpha } }}} \right)$$

The two Eqs. (15) and (16) represent the estimation of the *Qs* and *Ps* using both the stator flux and the rotor flux.

15 $$P_{s} = - \frac{3}{2}\frac{Lm}{{\sigma .Ls.Lr}}\mathop w\nolimits_{s} \left| {\mathop \Psi \nolimits_{s} } \right|\left| {\mathop \Psi \nolimits_{r} } \right|\sin (\lambda )$$

16 $$Q_{s} = - \frac{3}{2}\frac{ws}{{\sigma .Ls}}\left| {\mathop \Psi \nolimits_{s} } \right|\left( {\frac{M}{{\mathop L\nolimits_{r} }}\left| {\mathop \Psi \nolimits_{r} } \right|\cos (\lambda ) - \left| {\mathop \Psi \nolimits_{s} } \right|} \right)$$

In this proposed strategy, the reference rotor voltage values are expressed by the following equations:

17 $$\mathop V\nolimits_{dr}^{*} = \mathop y\nolimits_{1} (t) + \mathop y\nolimits_{2} (t)$$

With:

18 $$\left\{ \begin{gathered} y_{1} \left( t \right) = \beta_{1} \mathop {\left( {\left| {\mathop S\nolimits_{Qs} \left( t \right)} \right|} \right)}\nolimits^{{\mathop r\nolimits_{1} }} \cdot sign\left( {\mathop S\nolimits_{Qs} \left( t \right)} \right) + \beta_{2} \int {sign\left( {\mathop S\nolimits_{Qs} \left( t \right)} \right)dt} \hfill \\ y_{2} \left( t \right) = \beta_{3} \mathop {\left( {\left| {\mathop S\nolimits_{Qs} \left( t \right)} \right|} \right)}\nolimits^{{\mathop r\nolimits_{2} }} \cdot sign\left( {\mathop S\nolimits_{Qs} \left( t \right)} \right) + \beta_{4} \int {sign\left( {\mathop S\nolimits_{Qs} \left( t \right)} \right)dt} \hfill \\ \end{gathered} \right.$$

where, β_1_, β_2_, β_3_, and β_4_ are the gains of the proposed controller of the reactive power.

*r*_*1*_ and* r*_*2*_* are* the exponents defined for the proposed DSTSMC technique of reactive power (*r*_*1*_ = 1/2 for *y*_*1*_*(t)* and *r*_*2*_ = 1/3 for the *y*_*2*_*(t)*).

The reference value for quadrature rotor voltage is represented in Eq. (19).

19 $$\mathop V\nolimits_{qr}^{*} = \mathop y\nolimits_{3} (t) + \mathop y\nolimits_{4} (t)$$

With:

20 $$\left\{ \begin{gathered} \mathop y\nolimits_{3} (t) = \beta_{5} \mathop {\left( {\left| {\mathop S\nolimits_{Ps} \left( t \right)} \right|} \right)}\nolimits^{{\mathop r\nolimits_{3} }} \cdot sign\left( {\mathop S\nolimits_{Ps} \left( t \right)} \right) + \beta_{6} \int {sign\left( {\mathop S\nolimits_{Ps} \left( t \right)} \right)dt} \hfill \\ y_{4} \left( t \right) = \beta_{7} \mathop {\left( {\left| {\mathop S\nolimits_{Ps} \left( t \right)} \right|} \right)}\nolimits^{{\mathop r\nolimits_{4} }} \cdot sign\left( {\mathop S\nolimits_{Ps} \left( t \right)} \right) + \beta_{8} \int {sign\left( {\mathop S\nolimits_{Ps} \left( t \right)} \right)dt} \hfill \\ \end{gathered} \right.$$

where, β_5_, β_6_, β_7_, and β_8_ are the gains of the proposed controller of the active power.

*r*_*3*_ and* r*_*4*_* are* the exponents defined for the proposed DSTSMC technique of active power (*r*_*3*_ = 2/3 for *y*_*1*_*(t)* and *r*_*4*_ = 1/2 for the *y*_*2*_*(t)*).

*S*_*Qs*_ and *S*_*Ps*_ are the surfaces (errors) of the DFIG power (*S*_*Qs*_ =Qs*−Qs and *S*_*Ps*_ = Ps*−Ps).

**Figure 4:**
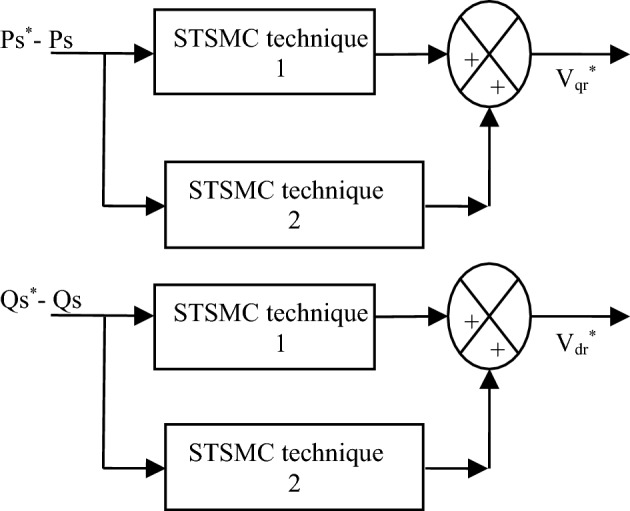
Proposed control scheme of the *Qs* and *Ps*.

Figure 4 represents the designed strategy for controlling the *Qs* and *Ps* of the DFIG using the DSTSMC technique, where two DSTSMC technique are used. The purpose of using DSTSMC technique is utilized in order to compute the reference values of both the quadrature and direct rotor voltages from the error in *Ps* and *Qs*.

## Simulation results

To study the characteristics of the proposed DPC technique based on DSTSMC controllers compared to the DPC-STSMC techniques, the numerical simulation of the DFIG-based MRWT system was accomplished using Matlab software. The parameter used is a 50 Hz, 380/690 V, p = 2, 1.5 MW, *fr* = 0.0024 Nm/s, *Rr* = 21 mΩ, *Lr* = 0.0136 H, *Rs* = 0.012 Ω, *J* = 1000 kg.m2, *Lm* = 13.5 mH, *Ls* = 13.7 mH^85,86^.

### Tracking test

**Figure 5:**
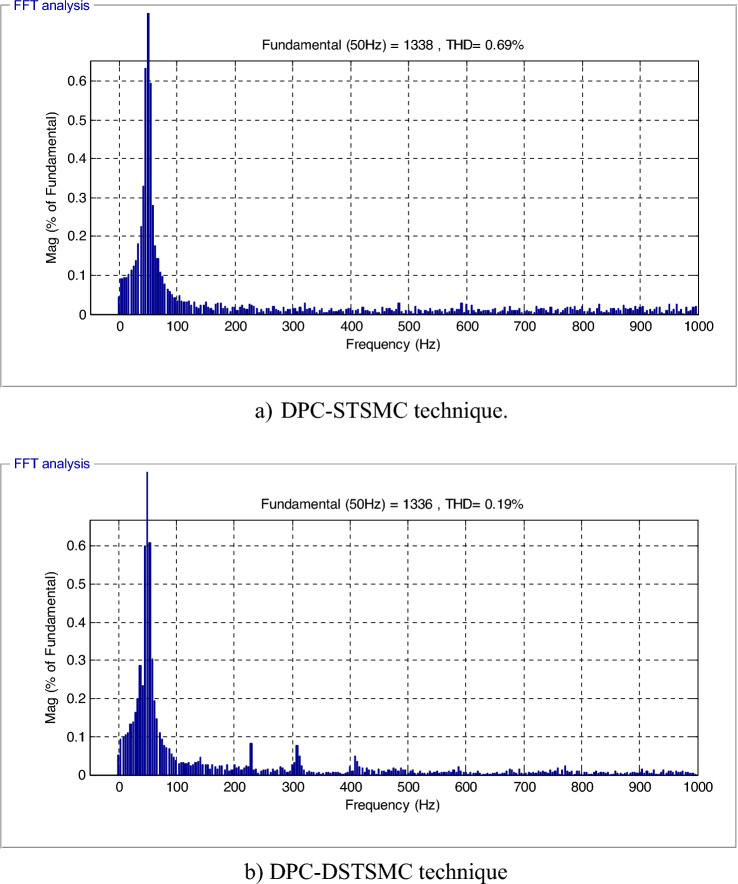
The THD value of the current.

The effectiveness of the proposed DPC-DSTSMC technique and the DPC-STSMC technique in reference tracking is examined in this section. From the results presented in Fig. 5a and 5b evident that the THD of generated currents for the DPC technique based on DSTSMSC strategies is minimized (0.69% for the traditional control and 0.19% for the proposed control), where the ratio of minimization was estimated at about 72.46% compared to the DPC-STSMC technique. On the other hand, it is noted that the DPC-DSTSMC technique provided a fundamental (50 Hz) of current signal amplitude of 1336 A and 1338 A for the traditional strategy. Therefore, the proposed DPC-DSTSMC technique provided lower amplitudes than the traditional strategy, which is an undesirable negative.

**Figure 6:**
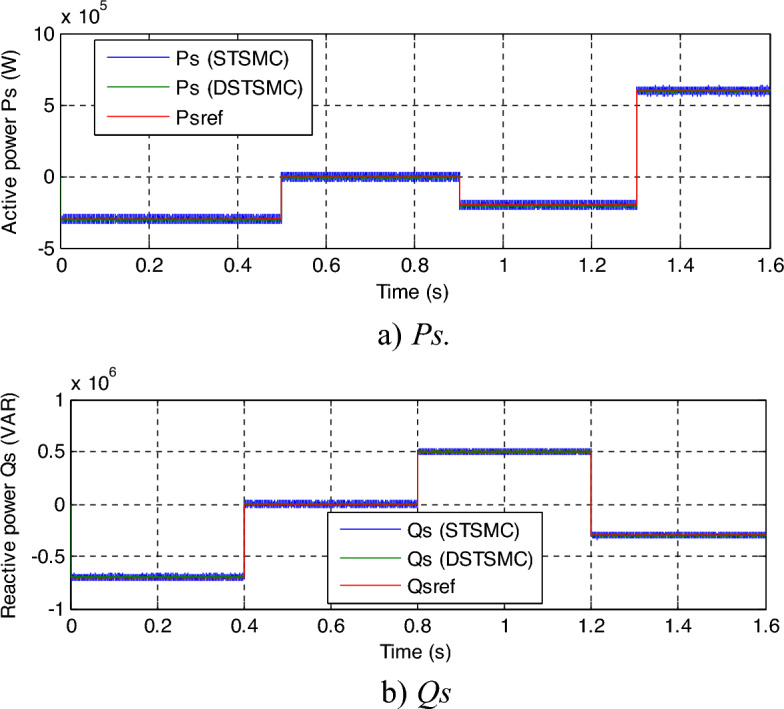
Results of the first test.

From the system responses given in Fig. 6a and b for both techniques the *Qs* and *Ps* tracks the reference powers without overshoot, with ripples in both *Ps* and *Qs*. These graphical results show the satisfactory performance of the two controllers in terms of tracking references. Table 1 presents the SSE values/ratios of the *Qs* and *Ps* of the DFIG. Through these ratios, the suggested control minimized the SSE of the *Qs* and *Ps* compared to the DPC-STSMC technique, where the minimization rates were estimated at 57.14% and 64.06% for each of the *Ps* and *Qs*, respectively.

**Table 1:** The ripples and SSE ratios/values of the *Qs* and *Ps* (first test).

	Techniques	*Ps*(W)	*Qs* (VAR)
SSE	DPC-STSMC	28,000	32,000
	DPC-DTSMC	12,000	11,500
	Ratios (%)	57.14	64.06
Ripples	DPC-STSMC	60,000	62,500
	DPC-DTSMC	10,000	20,000
	Ratios (%)	83.33	68

**Figure 7:**
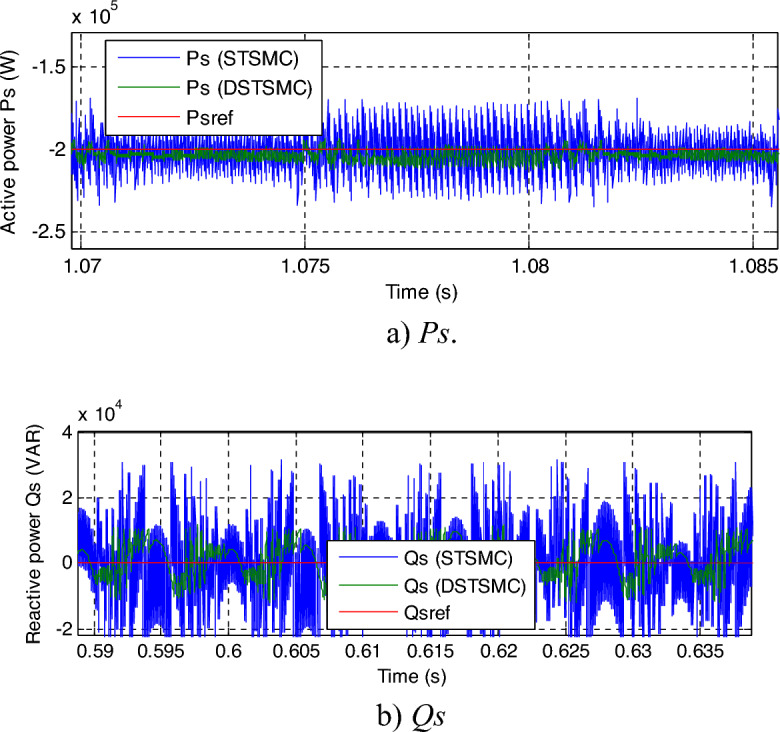
Zoom in the results of the first test.

The zoom in the *Qs* and *Ps* is shown in Fig. 7a and b, respectively. It can be seen that the DPC-DSTSMC technique reduced the undulations in *Ps* and *Qs* compared to DPC-STSMC technique. The ratios of ripples are recorded in Table 1. Through this table, the DPC-DSTSMC technique minimized the *Ps* and *Qs* ripples by 83.33% and 68%, respectively. So, through these high ratios, it can be said that the designed control is more efficient in minimizing undulations compared to the DPC-STSMC technique.

### Robustness test

**Figure 8:**
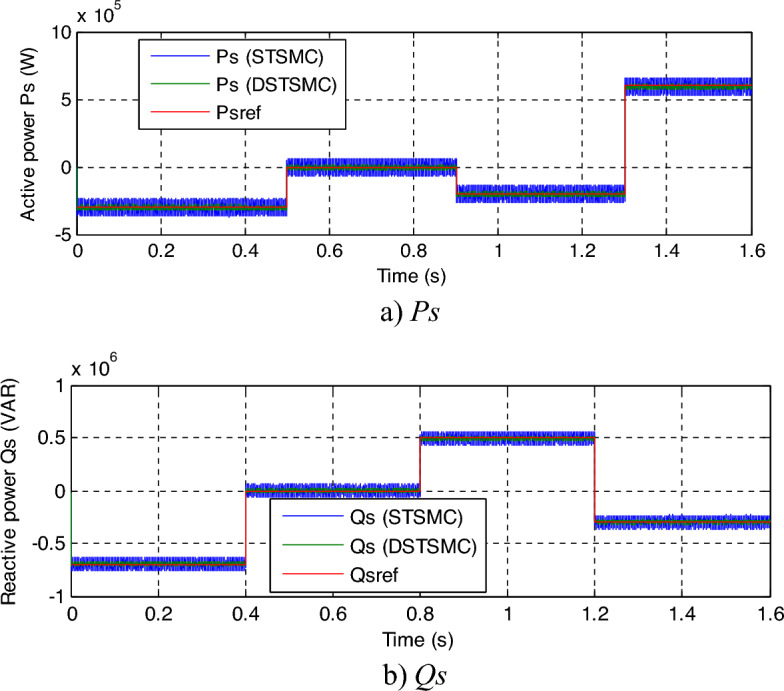
Results of the second test.

**Figure 9:**
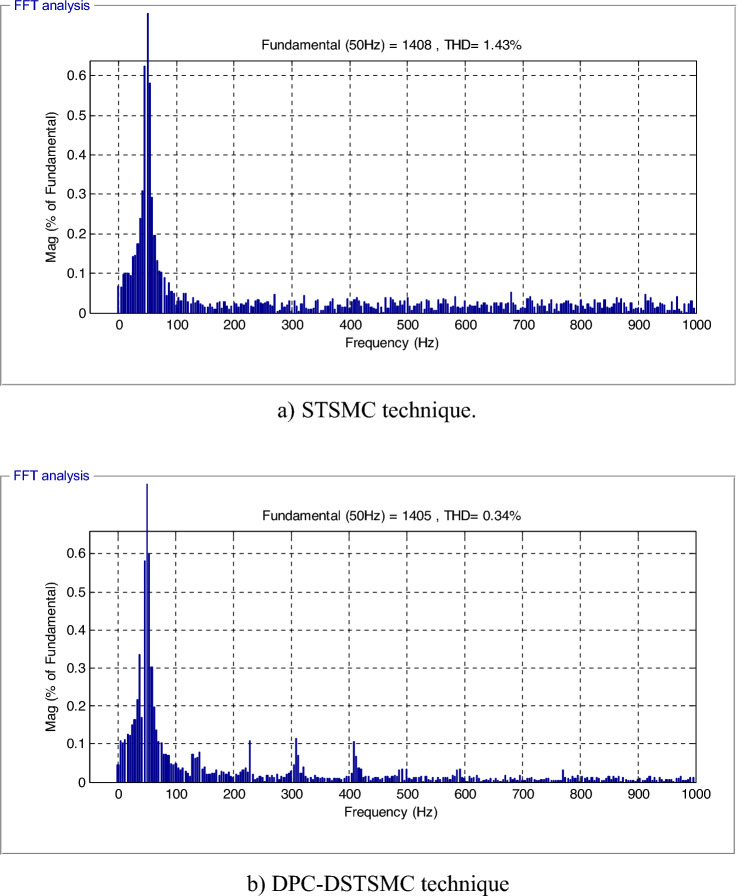
THD value of the current.

**Figure 10:**
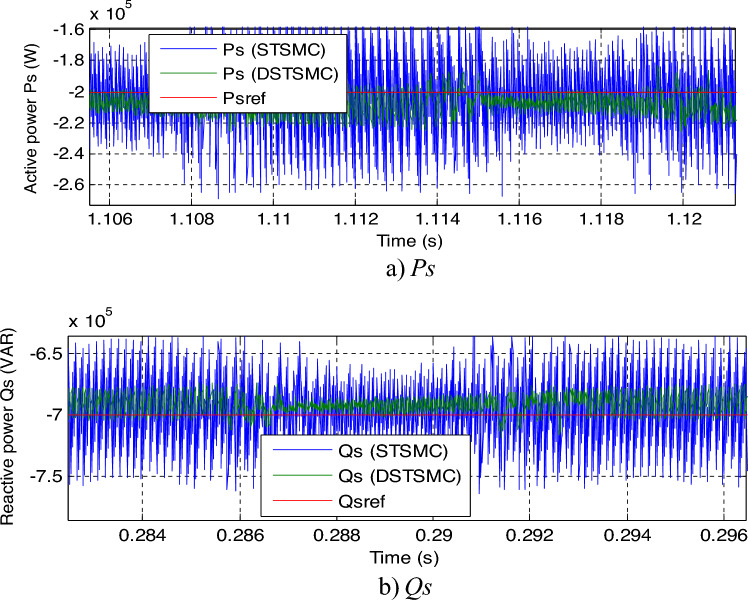
Zoom in the results of the second test.

In this section, the resistance values will be multiplied by 2 and the inductance values will be divided by 2. Numerical results are displayed in Figs. 8, 9 and 10. As presented by these figures, these variations and fluctuations present an apparent effect on the *Qs* and *Ps* curves, and the impact seems more noteworthy for the DPC-STSMC technique compared to the DPC-DSTSMC technique (Fig. 8a and b). Although the machine parameters have changed, the capabilities still follow the references well, with the proposed control having an advantage over the DPC-STSMC technique.

The THD of the current in the DPC-DSTSMC technique has been considerably reduced (Fig. 9a and 9b),where the value of THD of current was 1.43% and 0.34% for both the traditional and the proposed strategy, respectively. The proposed DPC-DSTSMC technique reduced the THD value by an estimated 76.22% compared to the DPC-STSMC method. Thus, it can be concluded that the designed DPC-DSTSMC technique is more robust than the DPC-STSMC techniques. Figure 9 also gives fundamental amplitude values (50 Hz) of the current of both strategies, where the amplitude is 1408 A for the traditional technique and 1405 A for the proposed strategy. Therefore, it can be said that the proposed strategy provided unsatisfactory results in terms of the amplitude of the fundamental signal (50 Hz) of current, and this can be attributed to the gain values, as this problem can be overcome by using smart strategies to calculate the gains of the proposed control.

In Fig. 10, a zoom is given of the results of the second test, as this figure gives a clear picture of the superiority of the proposed DPC-DSTSMC technique over the traditional DPC-STSMC strategy in terms of potential ripples. These ripples are less if the proposed DPC-DSTSMC technique is used, which is a positive thing that shows the superiority of the proposed DPC-DSTSMC technique over traditional control.

The SSE values ​​for each of the two designed techniques in this work are listed in Table 2, where the suggested strategy reduced the SSE value by an estimated 83.33% and 68.75% compared to the DPC-STSMC technique. The *Qs* and *Ps* ripples were reduced by using the DSTSMC technique compared to using STSMC controllers, where the minimization ratios were estimated at about 72.33% and 68.75% for each of the *Qs* and *Ps*, respectively.

**Table 2:** The ripples and SSE ratios/values of the *Qs* and *Ps* (second test).

	Strategies	*Ps* (W)	*Qs* (VAR)
SSE	DPC-STSMC	60,000	64,000
	DPC-DTSMC	10,000	20,000
	Ratios (%)	83.33	68.75
Ripples	DPC-STSMC	60,000	62,500
	DPC-DTSMC	10,000	20,000
	Ratios (%)	83.33	68

In Tables 3 and 4, a study is given on the extent to which the values of the THD of current and the amplitude of the fundamental signal (50 Hz) of current are affected, where the percentages of influence necessary to find the best control are calculated. From Table 3, it is noted that the THD value increased significantly in the second test for the two controls, which indicates that the THD value is affected by changing the DFIG parameters. This change was 51.47% and 44.11% for both the traditional and proposed strategies, respectively. Therefore, the proposed DPC-DSTSM strategy is less affected by the change in the THD value, which is a positive thing that indicates the robustness of the proposed DPC-DSTSM strategy. Regarding the amplitude shown in Table 4, it is noted that this amplitude increased in value in the second test for the two controls, and accordingly changing the DFIG parameters led to an increase in the amplitude of the fundamental signal (50 Hz) of current. This increase is estimated at 4.97% and 4.91% percent for both the traditional and proposed strategy, respectively.

**Table 3:** The ratios of change in the THD value between the two tests.

	THD value of current	
	DPC-STSMC technique	DPC-DSTSM strategy
Test_1	0.69%	0.19%
Test_2	1.43%	0.34%
Ratios	51.47%	44.11%

**Table 4:** Ratios change in the amplitude value of the fundamental signal (50 Hz) between the two tests.

	Amplitude value of the fundamental signal (50 H)	
	DPC-STSMC technique	DPC-DSTSM strategy
Test_1	1338 A	1336 A
Test_2	1408 A	1405 A
Ratios	4.97%	4.91%

### Third test

**Figure 11:**
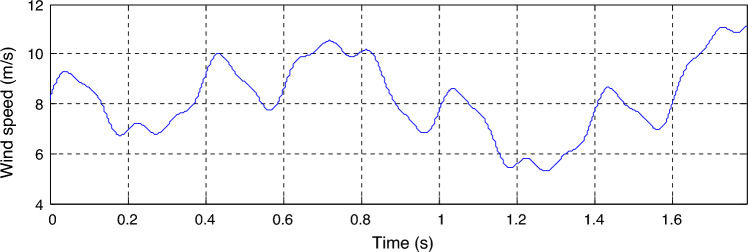
Wind speed profile.

**Figure 12:**
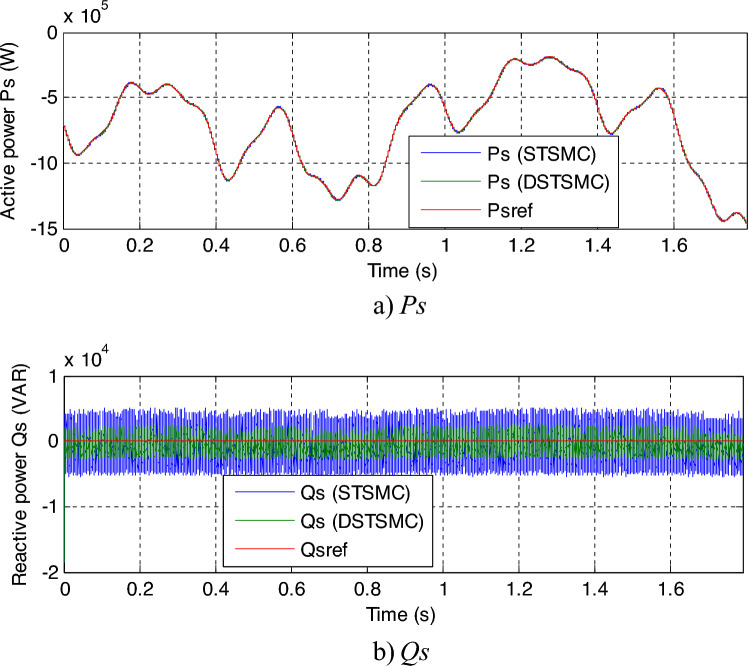
Results of the third test.

**Figure 13:**
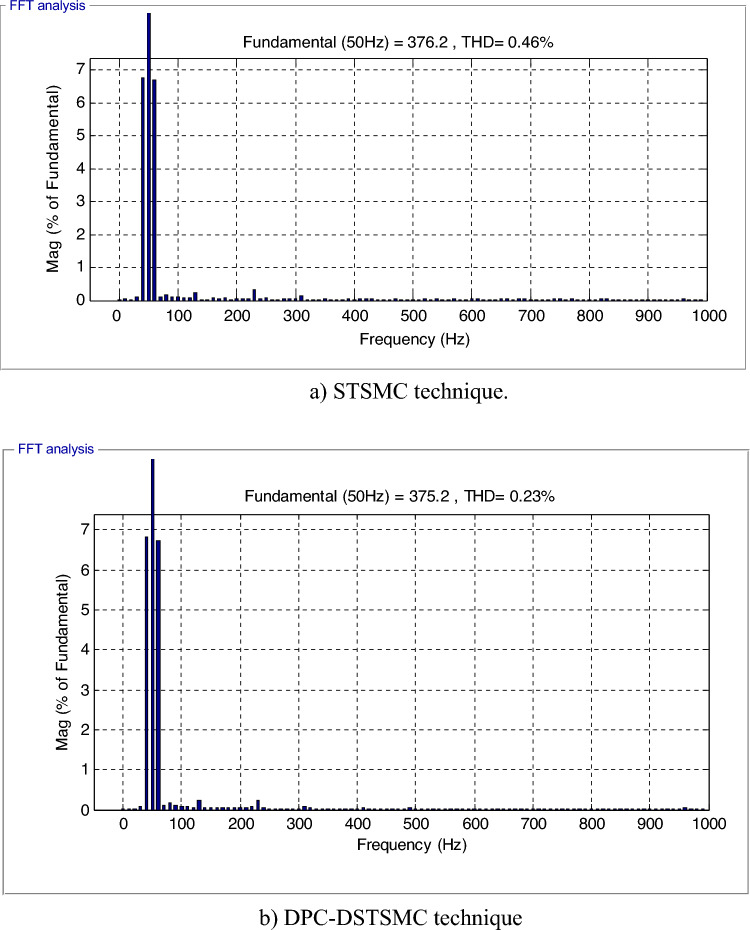
THD value of the current (Third test).

This test is different from the rest of the other tests, as a variable wind speed is used, as shown in Fig. 11. In this test, the MPPT strategy is used in order to determine the reference value for the *Ps*, and the reference value for the *Qs*is set at 0 VAR. The graphical results are shown. In Figs. 12 and 13, the numerical results are shown in Table 5. From Fig. 12a, it is noted that the *Ps* changes according to the change in wind speed. It is noted that as the speed increases, the value of the *Ps* increases and decreases as it decreases. But the *Qs* follows the reference value well and is not affected by the change in wind speed and remains a constant value (Fig. 12b), noting the presence of ripples in the power. In Fig. 13, the values of THD of current and amplitude of fundamental (50 Hz) signal of current of both techniques are shown. From this figure it is noted that the value of THD was 0.46% for the traditional DPC-STSM strategy and 0.23% for the proposed DPC-DSTSM strategy. So, the proposed DPC-DSTSM strategy reduced the THD value compared to the traditional DPC-STSM strategy, which is a positive thing that indicates that the quality of the current is better if the proposed DPC-STSM strategy is used, as the THD rate was reduced by an estimated 50% percent compared to the traditional DPC-STSM strategy. However, in terms of the amplitude value of the fundamental signal, the proposed DPC-DSTSM strategy provided unsatisfactory results, as the amplitude was estimated at 376.2 A and 375.2 A for both the traditional DPC-STSM strategy and the proposed DPC-DSTSM strategy, respectively.

**Table 5:** The SSE and ripples ratios/values of the *Qs* and *Ps* (Third test).

	Strategies	*Ps* (W)	*Qs* (VAR)
SSE	DPC-STSMC	3500	3350
	DPC-DTSMC	1800	1593.70
	Ratios	48.57%	52.42%
Ripples	DPC-STSMC	10,000	9883.80
	DPC-DTSMC	2500	4827
	Ratios	75%	51.16%

**Figure 14:**
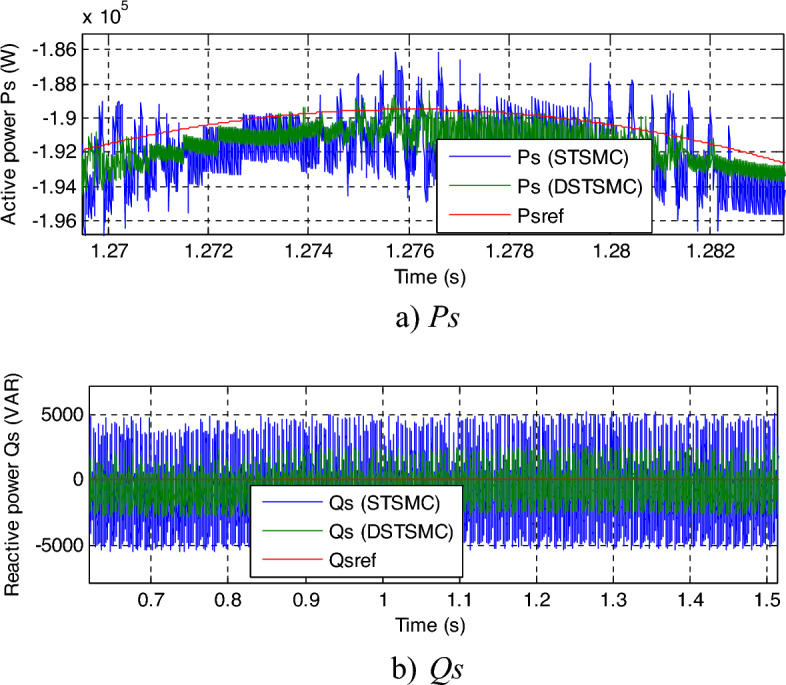
Zoom in the results of the second test.

In Fig. 14, the power ripples are shown. It is noted that the DPC-DSTSM strategy provided fewer ripples than the DPC-DSTSM strategy, which is a positive thing and indicates an effective performance in improving power quality compared to the traditional DPC-STSM strategy.

In Table 5, the obtained numerical results are given, where the values and percentages of reduction of SSE and ripples of DFIG power are presented. From this table, it is noted that the proposed DPC-DSTSMC technique has excellent results, and this is demonstrated by the calculated reduction rates. The proposed DPC-DSTSMC technique reduced the ripple value by percentages estimated at 75% and 51.16% for both *Ps* and *Qs*, respectively, compared to the traditional PC-STSMC technique. The reduction rates for SSE were estimated at 48.57% and 52.42% for both *Ps* and *Qs*, respectively, compared to conventional DPC-STSMC technique. So these high percentages indicate the effective performance in improving the characteristics of the studied energy system.

In Tables 6 and 7, the effect of the amplitude value of the fundamental signal (50 Hz) and the THD value of current of both techniques between the first and third tests is studied. In Table 6, it is noted that the THD value was affected and its value changed from the first test to the second test, where in the traditional DPC-STSMC technique the THD value decreased significantly. This decrease was estimated at 33.33%, which indicates that the traditional DPC-STSMC technique was greatly affected. In the proposed DPC-DSTSMC technique, it is noted that the THD value increased slightly from the first test to the third test, where this increase is estimated at 17.39%, which makes us say that the quality of the current was better in the first test compared to the third test.

**Table 6:** The ratios of change in the THD value between the first and third tests.

	THD value of current	
	DPC-STSMC technique	DPC-DSTSM strategy
Test_1	0.69%	0.19%
Test_3	0.46%	0.23%
Ratios	33.33%	17.39%

**Table 7:** Ratios change in the amplitude value of the fundamental signal (50 Hz) between the first and third tests.

	Amplitude value of the fundamental signal (50 H)	
	DPC strategy	DPC-NSTA technique
Test_1	1338 A	1336 A
Test_3	376.20 A	375.20 A
Ratios	71.88%	71.91%

In Table 7, the change in the amplitude of the fundamental signal (50 Hz) for the two controls in the first and third tests is shown. It is noted that this amplitude decreased in value significantly in the third test compared to the first test for the two controls. This decrease was estimated at 71.88% and 71.91% for both the traditional and proposed strategies, respectively. So the two controls provided the same reduction in amplitude, which shows that they have the same amount of effect. Also, using a variable wind speed affects the amplitude value significantly.

The designed technique reduced the THD of the supplied currents compared to other commands (Table 8). Based on the obtained results above, it can be stated that the proposed DPC-DSTSMC technique has proven its effectiveness in minimizing undulations and chattering problems, in addition to keeping the same characteristic of the DPC strategy.

**Table 8:** Comparison between others works.

References	Commands	HD (%)
^87^	Second-order SMC	3.13
^88^	DPC	4.88
	Virtual-flux DPC	4.19
^89^	DTC	7.83
^90^	Predictive torque control	2.15
Proposed techniques	DPC-STSMC	0.69
	DPC-DSTSMC	0.19

The proposed strategy is compared with some papers in terms of the reduction ratios of both undulations and SSE of *Ps* and *Qs*. The comparison results are listed in Tables 9 and 10, where it is noted that the DPC-DSTSMC technique provided high rates of ripples and SSE of *Ps* and *Qs* compared to some existing controls. So, it can be said that the DPC-DSTSMC technique has high performance and great effectiveness in improving the efficiency of the systems, and therefore it can be relied upon as a solution in the field of command.

**Table 9:** Comparison in terms of SSE for *Ps* and *Qs*.

References	SSE ratios	
	*Qs* (VAR)	*Ps* (W)
^91^	36.93%	35%
^92^	35.48%	62%
^93^	42.14%	47.57%
DPC-DSTSMC	51.85%	49.75%

**Table 10:** Comparison in terms of ripples minimization rates.

References			Ratios	
			*Qs (VAR)*	*Ps* (W)
^92^	Intelligent control		35%	36%
^93^	Backstepping control		46.93%	28.57%
^91^			36.93%	22.95%
^94^	STSMC		22.66%	21.75%
	Modified STSMC		21.23%	19.11%
^95^	Super-twisting algorithm		2.01%	7.35%
	Modified super-twisting algorithm		8.96%	13.44%
^68^	STSMC	Test_1	60.13%	33.33%
		Test_2	31.03%	33.33%
DPC-DSTSMC	First test		50.73%	34.25%
	Second test		51.85%	49.23%
	Third test		51.16%	75%

## Conclusions

In this new research paper, a novel robust control strategy is suggested to improve the characteristics of the DPC technique of the DFIG-MRWT systems. DSTSMC controller is designed to replace the traditional hysteresis comparators of the DPC strategy and PWM technique to control the RSC of DFIG. The novel designed DPC technique based on DSTSMC controllers preserves the characteristics of the DPC technique such as less parameter dependence and simplicity. The effectiveness and robustness of both strategies are studied under THD of current and power ripples. By comparing the characteristics of the suggested control scheme with the DPC-STSMC technique, it can be concluded that the DPC-DSTSMC technique has reduced the THD of current. The designed strategy has been very successful in improving the current quality provided by the DFIG-MRWT system. The results obtained from this work can be summarized in the following points:

- Power ripples are affected by changes in DFIG parameters.
- The reactive power ripple value was improved by 50.73%, 51.85%, and 51.16% in the three proposed tests compared to the traditional strategy. The active power ripples in the three tests were improved by 34.25%, 49.23%, and 75% compared to the conventional control.
- The value of power ripples is affected by the change in machine parameters and the shape of the wind speed change.
- The SSE value has been improved compared to the traditional control, where the reduction rates for active power were estimated at 83.33%, 57.14%, and 48.57% in the three proposed tests, and the reduction rates were estimated at 68.75%, 64.06%, and 52.47% for reactive power compared to the traditional strategy.
- The THD value was improved by 72.46%, 76.22%, and 50% in the three proposed tests compared to the traditional strategy.
- The amplitude value of the fundamental signal (50 Hz) is greatly affected by changing the machine parameters.

In future work, we will try to add intelligent techniques such as grey wolf optimization or neural networks to determine the values of the DPC-DSTSMC technique gains and compare the results with other strategies.

## Data Availability

The datasets used and/or analysed during the current study available from the corresponding author on reasonable request. In the event of communication, the corresponding author (Mourad Yessef, E-mail: mourad.yessef@usmba.ac.ma) will respond to any inquiry or request.

## Abbreviations

DFIG: Doubly-fed induction generator
STC: Super-twisting control
NNs: Neural networks
DSTSMC: Dual super-twisting sliding mode control
DPC: Direct power control
WE: Wind energy
MSVM: Modified space vector modulation
DTC: Direct torque control
EE: Electrical energy
MPPT: Maximum power point tracking
PWM: Pulse width modulation
PI: Proportional-integral controller
FL: Fuzzy logic
FOC: Field-oriented control
GA: Genetic algorithm
*Ps*: Active power
*Qs*: Reactive power
ST: Switching table
HC: Hysteresis comparator
THD: Total harmonic distortion
RSC: Rotor side converter
SMC: Sliding mode control
SSE: Steady-state error
GSC: Grid side converter
MRWT: Multi-rotor wind turbine system
STSMC: Super-twisting sliding mode control
SC: Synergetic control
TOSMC: Third-order sliding mode control
